# Supplementary material for: Diagnostic Findings of Transmissible Viral Proventriculitis Associated with Chicken Proventricular Necrosis Virus in Processed Broiler Chickens in Argentina
Source: Viruses. 2025 Apr 1;17(4):519. doi: 10.3390/v17040519 (PMC12031470; doi:10.3390/v17040519)
Supplement: Supplementary file 1 [file viruses-17-00519-s001.zip › viruses-3529430-supplementary.pdf]

Table S1. Farms involved on the 13 episodes of TVP-compatible grossly and condemned proventriculi at a broiler chicken processing plant in Argentina (Dec 2021-Apr 2022)

Farm number	Farm geolocation	Age of processed broilers (Days)	Average weight (Kg)	Processed broilers
**1**	-31.9949167 Lat<br>-58.3497777 Long	47	3.07	N/A
**2**	-32.18999522 Lat<br>-58.19608345 Long	N/A	N/A	N/A
**3**	-32.33049322 Lat<br>-58.20971761 Long	49	2.92	21.486
**4**	-32.00802012 Lat<br>-58.65552960 Long	42	2.97	126,143
**5**	-32.29521 Lat<br>-58.34004 Long	46	2.95	117,308
**6**	-32.19587809 Lat<br>-58.28020526 Long	50	2.94	61,293
**7**	-32.39559167 Lat<br>-58.33325833 Long	48	2.97	115,233
**8**	-32.06226141 Lat<br>-58.36849807 Long	48	2.85	32,041
**9**	-32.2760912 Lat<br>-58.26372809 Long	48	2.86	43,179
**10**	-32.0328667 Lat<br>-58.27524791 Long -	48	2.97	116,718
**11**	-32.2760912 Lat<br>-58.26372809 Long	48	2.79	29.641

Table S2. Histopathologic findings and scoring of TVP-compatible grossly condemned proventriculi at a broiler chicken processing plant in Argentina (Dec 2021-Apr 2022)

**Case number**							**Extension**	**Severity**	**Scores**
	LFA	NGE	H/MGE	DPG	ILII	PICT	F/M/D	Mi/Mo/S	0 to 4
1		X	X	X	X	X	D	S	4
2		X	X	X	X	X	D	S	4
3		X	X	X	X	X	D	S	4
4	X	X	X	X	X		D	S	3
5	X	X	X	X	X		D	S	3
6		X	X	X	X	X	D	S	4
7	X	X	X	X	X		D	S	3
8	X	X	X	X	X		M	Mo	3
9	X	X	X	X	X		D	S	3
10	X	X	X	X	X		D	S	3
11	X			X			M	Mi	2
12	X			X			M	Mi	2
13	X	X	X	X	X		D	S	3
14	X	X	X	X	X	X	D	S	4
15		X	X	X	X	X	D	S	4
16		X	X	X	X	X	D	S	4
17		X	X	X	X	X	D	S	4

**Case number**	LFA	NGE	H/MGE	DPG	ILII	PICT	**Extension** F/M/D	**Severity** Mi/Mo/S	**Grades** 0 to 4
18	X	X	X	X	X	X	M	S	4
19	X	X	X	X	X	X	M	S	4
20	X	X	X		X		M	S	3
21	X		X		X		M	Mi	2
22	X		X		X		D	Mi	2
23	X			X	X		D	Mi	2
24	X			X	X		M	Mi	2
25	X	X	X	X	X	X	M	S	4
26	X	X	X	X	X	X	M	S	4
27	X		X		X		D	Mi	2
28	X	X	X		X		M	Mo	3
29	X	X	X	X	X		M	Mo	3
30	X	X	X	X	X		D	Mo	3
31	X	X	X	X	X	X	D	S	4
32	X	X	X	X	X	X	D	S	4
33	X	X	X	X	X	X	D	S	4
34	X	X	X	X	X	X	D	S	4
35	X	X	X	X	X	X	D	S	4
36	X	X	X	X	X	X	M	S	4
37		X	X		X	X	M	Mo	3
38		X	X		X	X	M	Mo	3

Case number	LFA	NGE	H/MGE	DPG	ILII	PICT	Extension F/M/D	Severity Mi/Mo/S	Grades 0 to 4
39	X	X	X	X	X	X	D	Mo	3
40		X	X	X	X	X	D	S	4
41		X	X	X	X	X	M	S	4
42	X	X	X	X	X		M	Mo	3
43	X	X	X	X	X		D	Mo	3
44	X	X	X	X	X		M	S	4
45	X	X	X	X	X		D	S	4
46	X	X	X	X	X		D	S	4
47	X	X	X	X	X	X	M	S	4
48		X	X	X	X		M	Mo	3
49			X	X	X		M	Mo	3
50	X		X		X		M	Mi	2
51	X	X	X	X	X		D	Mi	2
52	X	X	X	X	X	X	D	S	4
53	X		X	X	X	X	D	S	4
54	X		X	X	X	X	D	S	4
55	X	X	X	X	X	X	D	S	4
56	X	X	X	X	X		M	S	4
57	X	X	X	X	X		D	Mo	3
58	X	X	X	X	X		D	S	4
59	X	X	X	X	X		M	S	4

**Case number**							**Extension**	**Severity**	**Grades**
	LFA	NGE	H/MGE	DPG	ILII	PICT	F/M/D	Mi/Mo/S	0 to 4
60	X	X	X	X	X	X	M	S	4
61		X	X	X	X		D	Mo	3
62		X	X	X	X	X	D	S	4
63		X	X		X	X	D	S	4
64		X			X	X	M	S	4
65		X		X	X	X	D	Mo	3
66	X	X	X		X	X	D	S	4
67	X	X	X	X	X	X	M	S	4
68	X	X	X	X	X	X	D	S	3
69	X	X	X	X	X	X	M	S	4
70	X	X	X	X	X		M	Mo	4
71	X	X	X	X	X		M	Mo	4
72	X	X	X	X	X		D	Mo	4
73	X	X	X	X	X		M	S	4
74	X	X	X	X	X		D	Mo	4
75	X	X	X	X	X	X	D	S	4
76	X	X	X	X	X	X	M	S	4
77	X	X	X	X	X		M	S	4
78	X	X	X	X	X		D	S	4
79	X	X	X	X	X	X	D	S	4
80	X		X	X	X	X	M	S	4

**Case number**	LFA	NGE	H/MGE	DPG	ILII	PICT	**Extension** F/M/D	**Severity** Mi/Mo/S	**Grades** 0 to 4
81	X		X	X	X		M	Mo	2
82	X		X	X	X		M	Mo	2
83	X	X	X	X	X		M	Mi	2
84	X	X	X	X	X	X	M	Mi	2
85	X	X	X		X	X	D	Mi	2
86	X	X	X		X	X	D	S	4
87	X	X	X	X	X	X	D	S	4
88	X	X	X	X	X	X	D	S	4
89	X	X	X	X	X		D	S	4
90	X	X	X	X	X		D	S	4
91	X	X	X	X	X	X	M	Mo	4
92	X	X	X	X	X	X	D	Mo	3
93	X	X	X	X	X	X	D	S	4
94	X	X	X	X	X	X	D	S	4
95	X	X	X	X	X	X	D	Mo	3
96		X	X	X	X	X	D	S	4
97		X	X	X	X	X	D	S	4
98		X	X	X	X	X	D	S	4
99	X	X	X	X	X	X	D	S	4
100		X	X	X	X		D	S	4
101		X	X	X	X	X	M	S	4

Case number	LFA	NGE	H/MGE	DPG	ILII	PICT	**Extension** F/M/D	**Severity** Mi/Mo/S	**Grades** 0 to 4
102	X	X	X	X	X	X	D	Mo	3
103		X	X	X	X	X	D	S	4
104		X	X	X	X	X	D	S	4
105	X	X		X	X	X	D	S	4
106	X			X	X	X	D	S	4
107	X		X	X	X		D	S	4
108		X	X	X	X		M	S	4
109	X	X	X	X	X		M	Mi	2
110		X	X	X	X		D	Mo	3
111		X	X	X		X	M	S	4
112		X	X	X		X	D	Mo	4
113	X	X	X	X	X		D	S	4
114		X	X	X	X	X	M	S	4
115	X	X	X	X	X	X	D	S	3
116	X	X	X	X	X	X	D	S	4
117		X	X	X	X		D	S	4
118	X	X	X	X	X		M	S	4
119	X	X	X	X	X		D	Mo	3
120	X		X	X	X		D	S	4
121	X		X	X	X		D	S	4
122	X		X		X		D	S	4

LFA = Lymphocytic follicular aggregates // NGE = Necrosis of glandular epithelium // H/MGE = Hyperplasia/metaplasia of glandular epithelium // DPG = Distension of proventricular glands // ILII = Intraglandular lymphocytic inflammatory infiltration // PICT = Proliferation of interstitial connective tissue // F/M/D = Focal / Multifocal / Diffuse // Mi/Mo/S = Mild / Moderate / Severe

Supplemental Table S3. Episodes of TVP-compatible grossly and condemned proventriculi at a broiler chicken processing plant in Argentina (Dec 2021-Apr 2022) including histopathologic scores and CPNV RT-PCR results

Episode of condemnation	Condemned proventriculi number (FFPE block number)	Histopathologic score	CPNV RT-PCR result per block number
1	1 (1)	4	(-)
	2 (1)	4	
	3 (1)	4	
2	4 (2)	3	(+)
	5 (2)	3	
	6 (3)	4	(+)
	7 (3)	3	
	8 (4)	3	(+)
	9 (4)	3	
	10 (4)	3	
3	11 (5)	2	(+)
	12 (5)	2	
	13 (6)	3	(+)
	14 (6)	4	
	15 (7)	4	(+)
	16 (7)	4	
	17 (7)	4	
	18 (8)	4	(+)
	19 (8)	4	
	20 (8)	3	

4	21 (9)	2	(+)
	22 (9)	2	
	23 (10)	2	(+)
	24 (10)	2	
	25 (11)	4	(+)
	26 (11)	4	
	27 (12)	2	(+)
	28 (12)	3	
	29 (13)	3	(+)
	30 (13)	3	
**Episode of condemnation**	**Condemned proventriculi number (FFPE block number)**	**Histopathologic score**	**CPNV RT-PCR result**
5	31 (14)	4	(+)
	32 (14)	4	
	33 (15)	4	(+)
	34 (15)	4	
	35 (16)	4	(+)
	36 (16)	4	
	37 (17)	3	(+)
	38 (17)	3	
6	39 (18)	3	(+)
	40 (18)	4	
	41 (19)	4	(+)
	42 (19)	3	
	43 (19)	3	
	44 (19)	4	
	45 (20)	4	(+)
	46 (20)	4	
	47 (21)	4	(+)
	48 (21)	3	
	49 (22)	3	(+)
	50 (22)	2	
	51 (23)	2	(-)
	52 (23)	4	
7	53 (24)	4	(+)
	54 (24)	4	
	55 (25)	4	(+)
	56 (25)	4	
	57 (26)	3	(-)
	58 (26)	4	
	59 (27)	4	(+)
	60 (27)	4	

8	61 (28)	3	(+)
	62 (28)	4	
	63 (29)	4	(+)
	64 (29)	4	
	65 (30)	3	(+)
	66 (30)	4	
	67 (31)	4	(+)
	68 (31)	3	
	69 (32)	4	(+)
	70 (32)	4	
**Episode of condemnation**	**Condemned proventriculi number (FFPE block number)**	**Histopathologic score**	**CPNV RT-PCR result**
9	71 (33)	4	(-)
	72 (33)	4	
	73 (34)	4	(-)
	74 (34)	4	
	75 (34)	4	
	76 (35)	4	(+)
	77 (35)	4	
	78 (36)	4	(+)
	79 (36)	4	
	80 (37)	4	(-)
	81 (37)	2	
10	82 (38)	2	(+)
	83 (38)	2	
	84 (39)	2	(+)
	85 (39)	2	
	86 (40)	4	(+)
	87 (40)	4	
	88 (41)	4	(+)
	89 (41)	4	
	90 (42)	4	(+)
	91 (42)	4	
11	92 (43)	3	(+)
	93 (43)	4	
	94 (44)	4	(-)
	95 (44)	3	
	96 (45)	4	(+)
	97 (45)	4	
	98 (46)	4	(+)
	99 (46)	4	
	100 (47)	4	(+)
	101 (47)	4	

	102 (48)	3	(+)
	103 (48)	4	
	104 (49)	4	(-)
	105 (49)	4	
12	106 (49)	4	
	107 (50)	4	(+)
	108 (50)	4	
	109 (51)	2	(+)
	110 (51)	3	
	111 (52)	4	(-)
	112 (52)	4	
**Episode of condemnation**	**Condemned proventriculi number (FFPE block number)**	**Histopathologic score**	**CPNV RT-PCR result**
	113 (53)	4	(+)
	114 (53)	4	
	115 (54)	3	(+)
	116 (54)	4	
13	117 (55)	4	(+)
	118 (55)	4	
	119 (56)	3	(+)
	120 (56)	4	
	121 (57)	4	(+)
	122 (57)	4	
